# The Follicular Output Rate (FORT) as a method to evaluate transdermal testosterone efficacy in poor responders

**DOI:** 10.5935/1518-0557.20200086

**Published:** 2021

**Authors:** Roser Solernou, Sara Peralta, Gemma Casals, Marta Guimera, Marina Solsona, Aina Borras, Dolores Manau, Francesc Fàbregues

**Affiliations:** 1 Institute Clinic of Gynecology, Obstetrics and Gynecology. Hospital Clinic. Barcelona, Spain

**Keywords:** Follicular Output Rate, Transdermal testosterone, Poor responder, ovarian stimulation

## Abstract

**Objective:**

Follicular Output Rate (FORT) is an efficient quantitative and qualitative marker of ovarian responsiveness to gonadotropins. Transdermal testosterone (TT) has been used as adjuvant therapy to gonadotrophins in order to improve ovarian response in poor responders (PR). The aim of this study was to analyze whether TT can improve follicular sensitivity to gonadotropins using FORT.

**Methods:**

This retrospective study, held in a tertiary-care university hospital included 90 PR patients, according to the Bologna criteria. Patients in Group 1 (n = 46) received transdermal application of testosterone preceding gonadotrophin ovarian stimulation under pituitary suppression. In Group 2 (n = 44) ovarian stimulation was carried out with high-dose gonadotrophin in association with minidose GnRH agonist protocol. We analyzed ovarian stimulation parameters and IVF outcomes. We determined antral follicle count (AFC) (3-8 mm) before ovarian stimulation, pre-ovulatory follicle count (PFC) (16-22 mm) and the day of hCG administration. We calculated the FORT using the PFCx100/AFC ratio.

**Results:**

Baseline characteristics and ovarian reserve parameters were similar in both groups. FORT and oocytes retrieved were significantly higher in group 1 vs group 2. There were no significant differences in pregnancy rates. In group 1 there was a significant correlation between FORT and AFC.

**Conclusions:**

This study suggests that the potential beneficial mechanism of TT in poor responder patients may be based on increasing the antral follicle sensitivity to gonadotrophin. FORT is an excellent tool to demonstrate this.

## INTRODUCTION

The management of poor responders (PR) is a major challenge in IVF. Various stimulation regimens and interventions have been proposed for improving pregnancy outcomes in PR. These also include several adjuvant therapies such as androgen supplements (dehidroepiandrosterone and testosterone) and GH (Ubaldi et al. 2014, Ata & Seli, 2015, Kamath et al., 2019). Most of the Randomized Clinical Trials (RCT) evaluating these clinical add-ons had poorly reported methodologies, small sample sizes, or were at risk of bias. The conclusions for almost all the clinical adjuncts is the need for robust, well-designed RCTs to address both clinical and cost-effective parameters of the interventions. Although all of these adjuvant therapies provided promising results in initial studies, their efficacy has not been solidly demonstrated in RCT studies. The challenge in conducting an adequately powered RCT is the sample size. To show an improvement of 5% in LBR with 80% power after an intervention, a sample size of more than 2,000 participants is needed, which would be difficult to recruit even in large centers (Kamath et al., 2019).

According to results obtained in animals, it is possible that androgens exert a facilitating role in follicle responsiveness to FSH, which leads to an increase in the number of growing follicles (Weil *et al*., 1999; Vendola *et al*., 1998; 1999). A simple method to evaluate these effects is controlled ovarian hyperstimulation (COH); however, ovarian response to COH is quantitatively influenced by the pretreatment amount of small antral follicles.

To overcome this difficulty, an objective index to assessing antral follicle responsiveness to exogenous FSH, the Follicular Output Rate (FORT) is being developed (Genro *et al*., 2011; Gallot *et al*., 2012; Grynberg & Labrosse, 2019). FORT is calculated based on the ratio between the number of preovulatory follicles obtained in response to FSH administration and the preexisting pool of small antral follicles. Hence, in case of androgen pretreatments, FORT constitutes an interesting clinical strategy to check whether the ability of antral follicles to respond to exogenous gonadotrophins has been improved (Fanchin *et al*., 2011).

The aim of this study was precisely to assess whether transdermal testosterone was able to improve FORT in poor responders.

## MATERIALS AND METHODS

### Patients

This study was performed by a retrospective analysis of our database of women referred to our center for IVF, and was conducted from January 2013 to May 2016 in the Assisted Reproduction Unit of the Hospital Clinic in Barcelona (Spain). Before starting the IVF cycle, the patients were suggested to use Transdermal testosterone (TT) as an adjuvant therapy to ovarian stimulation. Two patients rejected this therapy. Review Board and informed consent was obtained from all individual participants, included in the study (HB-15-EL-RS-C). We recruited 90 poor responder patients according to the Bologna criteria (Ferraretti *et al*., 2011). All the patients were in good normal thyroid, kidney and hepatic health laboratory results, and they had regular menstruation periods with a 21-35 days duration. None of them had taken any infertility medication in the 3 months prior to the study.

**Figure 1:**
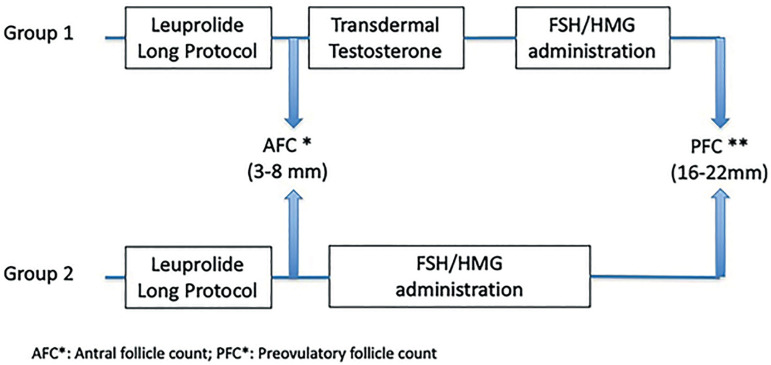
Schematic representation protocol

We used the long GnRh agonist protocol in all the patients. Group 1 patients were treated with TT preceding ovarian stimulation with gonadotropins, and Group 2 started ovarian stimulation after ovarian arrest was confirmed. (Fig 1).

**Figure 2:**
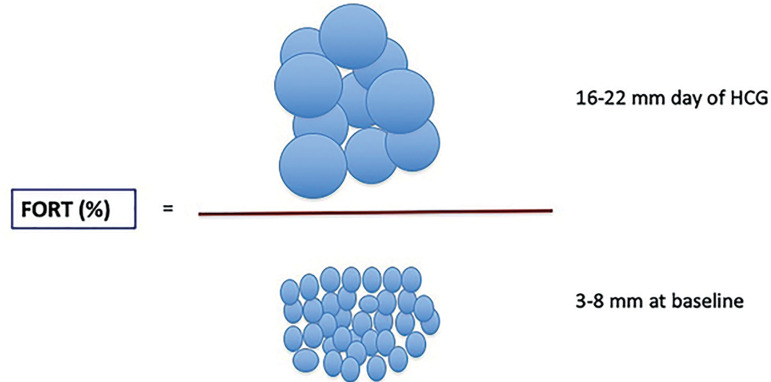
Follicular output rate= Ratio of pre-ovulatory follicle count on day hCG x 100/antral follicle count

FORT was calculated as the ratio of pre-ovulatory follicle count (PFC)(16-22 mm in diameter) on dhCG x 100/small antral follicle (3-8 mm in diameter) count at baseline (ovarian arrest day) (Fig 2). We evaluated the following study parameters: days of stimulation, pre-ovulatory follicles on the day of human chorionic gonadotropin (hCG) administration, number of oocytes retrieved, number of embryos achieved and transferred. We also assessed pregnancy outcomes, including clinical and ongoing pregnancy rates.

There was no perimplantation diagnosis in any of the cycles

### Stimulation regimens

We suppressed the pituitary suppression by s.c administration of leprolide acetate (Procrin; Abbott Laboratories, Madrid, Spain). This treatment was started in the midluteal phase of the previous cycle and given 1 mg daily, then reduced to 0.5 mg after ovarian arrest was confirmed, and continued until the administration of HCG. 

Group-1 patients started TT on the day when pituitary suppression was confirmed and the therapy was continued for 5 days. Transdermal testosterone treatment was carried out using a daily single patch with a 2.5 mg/day nominal delivery rate of testosterone (Testopatch, Pierre Fabre Iberica SA, Barcelona, Spain), which was applied to the thigh at night and removed always at 09:00h in the morning.

This transdermal delivery system maintains testosterone levels stable within narrow ranges with little within - and between - subject variation, providing a highly controllable and reliable way of delivering testosterone, and the hormonal dose administered can be modified according to the patch duration (Buckler *et al*., 1998; De Sanctis *et al*., 1998; Mazer, 2000). We chose to use 20 mg/kg of testosterone per day for 5 days, based on previous experimental studies in primates (Vendola *et al*., 1998; 1999). Thus, in each patient, the patch was applied at night, supposedly left in place for a predetermined number of hours in order to provide the desired daily dose of testosterone (e.g. in a woman weighing 60kg and needing 1,200mg/day, the patch was used for 12h [0.1mg/h delivery rate 12h. 1.2mg or 1200mg], and applied at 21:00h). We performed the testosterone therapy according to a routinely used protocol (Balasch *et al*., 2006; Fàbregues *et al*., 2009; 2019).

In Group 2 gonadotropin therapy started after ovarian arrest was confirmed. In both groups controlled ovarian hyperstimulation was performed with 300IU per day of r-hFSH (Gonal-F, Merck S.A., Madrid, Spain) together with 75IU HMG (Menopur, Ferring S.A., Madrid, Spain). The criteria for hCG administration (250mg s.c.Ovitrelle, Serono S.A.) were the presence of two or more follicles >18 mm in diameter, with >4 follicles measuring >14mm in association with a consistent rise in serum E2 concentration.

The cycle was cancelled when there were less than 3 follicles with diameter >14 mm after 8-9 days of gonadotropin therapy, or after 4-5 additional treatment days without attaining, or the imminent prospect of attaining, the criteria for hCG administration.

Oocyte aspiration was performed under vaginal ultrasonography 35-36h after hCG administration. Embryo grading was recorded according to published criteria (Veeck, 1999); embryos graded 1 or 2 were considered of high quality. In both groups, embryo transfer was performed in the cleavage stage (day 3). The luteal phase was supported with vaginal micronized progesterone (600mg/day given at 8h intervals) starting on the day following oocyte aspiration and continuing either up to menstruation or, if the patients became pregnant, for at least the first 3 weeks of pregnancy.

Pregnancy was diagnosed by a positive serum β-hCG test 12 days after ET. Clinical pregnancy was defined by observation of a fetal heartbeat using transvaginal ultrasonography upon 5-6 weeks of gestation.

### Statistical analysis

Sample size was based on two previous studies from our group, showing that: (i) multifollicular development and oocyte retrieval was obtained in 80% of previously low responders receiving pretreatment with TT and gonadotrophin ovarian stimulation as used in patients in Group 1 (Balasch *et al*., 2006); and (ii) 46% of patients having their first cycle of IVF cancelled because of poor follicular response underwent oocyte retrieval in the second IVF attempt, when treated according to the approach used in Group 2 (Peñarrubia *et al;* 2005). The sample size required to provide an 80% power to detect this magnitude of treatment effect between groups was calculated to be a minimum of 31 patients per group, using a two-tailed analysis with a detection limit of 5%, to avoid a type I error in hypothesis testing.

All statistical analyses were performed using the SPSS version 23.0 software (Chicago, IL, USA). We used a t-test to compare the mean values between two different stimulation protocols. Differences in outcome rates were analyzed using an χ2 test or the Fisher’s exact test. p<0.05 was considered statistically significant. The Spearman’s test was used to determine the correlation between FORT and ovarian reserve parameters in patients treated with TT.

## RESULTS

As summarized in Table 1 the groups were similar with respect to age, body mass index (BMI), duration of infertility, infertility causes, antral follicle count (AFC), Anti-Müllerian hormone (AMH) levels and basal FSH and estradiol. There were no reported major side effects after testosterone therapy, and two protocols were well tolerated by all patients.

**Table 1:** Baseline characteristics of poor-responder IVF patients in Groups 1 (TT) and 2 (No TT)

Variable	Group 1 (n = 46)	Group 2 (n = 44)
**Age (years)**	36.8 ± 0.3	37 ± 0.3
**BMI (Kg/m2)**	24 ± 0.5	24.2 ± 0.7
**Infertility factor** **Male factor (n,%)** **Unexplained (n,%)** **Tubal factor (n,%)** **Endometriosis (n,%)**	21 (45.6) 11 (23.9) 9 (19.5) 5 (10.8)	18 (40.9) 10 (22.7) 10 (22.7) 6 (13.6)
**Duration of infertility (years)**	5.8 ± 2.7	5.6 ± 2.2
**Basal FSH (IU/l)**	12.3 ± 0.5	13.2 ± 0.2
**Antral follicle count (n)**	5.4 ± 0.4	5.8 ± 0.3
**AMH (ng/ml)**	0.7 ± 0.3	0.9 ± 0.1

Values are mean ± SD. Between group differences all non-significant at a 0.05 level of significance.

Table 2 shows stimulation parameters and IVF outcomes, and we found that the number of cancelled cycles was similar in both groups (10.9 vs 22.8 %). The duration of ovarian stimulation was significantly shorter in Group 1 when compared with Group 2 (10.2±0.3 vs 11.5±0.2 days). FORT and number of oocytes retrieved were also significantly higher in Group 1 vs Group 2 (73±4.6 vs 55.6±4.1 %); and (4±0.3 vs 3±0.3), respectively. There were no differences in fertilization, implantation and pregnancy rates in both groups.

**Table 2:** Ovarian response, ovum retrieval and IVF/ICSI outcomes in Groups 1 and 2

Variable	Group 1	Group 2	*p*-value
**Patients with hCG and ovum retrieval (n,%)**	41 (89.1)	34 (77.2)	0.3
**Days of ovarian stimulation**	10.2 ± 0.3	11.5 ± 0.2	0.01
**Pre-ovulatory follicle (16-22mm)**	4.1 ± 1.2	3 ± 0.9	0.2
**FORT (%)**	73 ± 4.6	55.6 ± 4.1	0.01
**No. of oocytes retrieved^a^**	4 ± 0.3	3 ± 0.3	0.04
**No. of metaphase II oocytes^a^**	4 ± 0.3	2.8 ± 1.8	0.15
**No. of 2PN on day 1^a^**	3.9 ± 0.1	2.4 ± 0.1	0.1
**No. of embryos obtained^a^**	2.6 ± 0.3	1.7 ± 0.1	0.29
**No of embryos transferred^a^**	1.7 ± 0.5	1.4 ± 0.4	0.28
**Implantation rate (%)^a^**	28.3	22.8	0.58
**Clinical pregnancy** **Number** **Per oocyte retrieval (%)** **Twins (n,%)** **Miscarriages (n, %)**	15 36.5 1 1	10 29.4 0 1	N.S N.S N.S N.S

Values are mean ± SD.

a Values are relative to the number of patients with oocyte retrieval.

When we evaluate the correlations between FORT and ovarian reserve parameters in Group-1 patients, there was a significant correlation with AFC (r=0.41; *p*=0.001). However, this was not demonstrated with age, basal FSH and AMH levels.

## DISCUSSION

To our knowledge, this is the first study to analyze the value of FORT in poor responder patients, and specifically in those who have undergone adjuvant treatment with TT in IVF cycles.

The Cochrane review on the role of testosterone pretreatment included four RCTs with poor responders (Nagels *et al.,* 2015). Pooling the RCTs showed that pretreatment with testosterone was associated with improved LBR (OR 2.60; 95% CI, 1.30-5.20; 4 RCTs, n =345). However, a sensitivity analysis removing the studies at high risk of performance bias showed no statistically significant difference in LBR between the testosterone and control groups (OR 2.00; 95% CI, 0.17-23.49; 1 RCT, n= 53). These contradictory results could be explained by the different doses used (10 to 12 mg/daily), the way of application (patches or gel) and the pretreatment length (5 to 21 days) (Massin *et al*., 2006; Fàbregues *et al*., 2009;^2019^; Kim *et al*., 2011; Doan *et al.*, 2017). Obviously, only a multicenter randomized study with a large number of patients can definitively clarify the true efficacy of this add-on therapy in ovarian stimulation (Polyzos *et al*., 2018).

The above notwithstanding, in this study we chose to use TT for 5 days on the basis of studies in primates (Vendola *et al*., 1999), and also reports from previous clinical studies (Balasch *et al*., 2006; Fàbregues *et al.*, 2009; 2013; 2019). The effects of T on follicular response appears to be mediated by increasing FSH-receptor activity and by stimulating insulin-like growth factor I (IGF-I) (Weil *et al*.,1999). In studies in subhuman primates, androgen receptor gene expression correlated with follicle growth, and the T treatment significantly increased granulosa cell (GC) FSH receptor messenger RNA (mRNA) (Vendola *et al*., 1998). In this line, previous studies have shown that just 5 days of T caused a progressively increased level of circulating IGF-I throughout the ovarian stimulation for more than a week after TT was discontinued, and it has been suggested that increased levels of IgF1 could amplify the effect of gonadotropins in the ovary (Balasch *et al*., 2006; Meldrum *et al*., 2013).

Regardless of the controversy that exists regarding the clinical evidence of the androgen therapy usefulness, it is especially interesting to analyze whether there really is a greater capacity of androgens for the sensitization of follicles to the action of gonadotropins. In this sense, FORT is an excellent method to evaluate it (Fanchin *et al*., 2011).

Interestingly, the number of pre-ovulatory follicles obtained at the end of controlled ovarian hyperstimulation (COH) is not a reliable reflection of antral follicle sensitivity to FSH, as it is greatly influenced by the number of the small antral follicles available before treatment. This contingency constitutes a possible explanation for the inconstant relationship between the absolute counting of growing follicles obtained in COH and IVF-ET outcomes (Fanchin *et al*., 2011; Grynberg & Labrosse, 2019; Besow *et al*., 2019).

Several studies have established FORT groups according to tercile values: low (<42%), average (42-58%) and high (>58%) in order to analyze the effective response to FSH and ovarian follicular competence (Gallot *et al.*, 2012; Tan *et al*., 2019). According to the data from this study, the observed FORT can be considered medium-high (73 ± 4.6 vs 55.6 ± 4.1), and this is in agreement with previous studies in the sense that with ovarian aging, antral follicles do not lose their aptitude to respond to FSH, and probably reveals a compensating mechanism for preserving ovulatory folliculogenesis. (Gallot *et al*.,2012).

Taking into account the homogeneity of the studied groups, the significantly higher FORT in Group 1 vs 2 of the study has special relevance. This could also be related to a shorter duration on the days of stimulation and an increase in the number of oocytes obtained. This data agrees with those obtained in other studies (Gallot *et al*., 2012; Griesinger *et al*., 2019).

In accordance with previous studies, a correlation between FORT and antral follicle count was also shown in our study (Gallot *et al*., 2012); however, with AMH levels we did not find an aspect that was reported in other studies (Genro *et al*., 2011). It has been speculated that the negative correlation between AMH levels with FORT could be related to the possible inhibitory effect of this hormone with the sensitivity of follicles to FSH. However, this aspect has been suggested in patients with normal ovarian reserve but not in low responders.

TT has been used in poor responders with long GnRH agonist and antagonist protocols, finding no differences in terms of pregnancy rate or live birth rate (Fábregues *et al*., 2019). In this study, we included patients in whom the long agonist protocol was used based on the study sample size being calculated for this protocol.

The main limitation of this study was its retrospective design and small sample size. However, the poor responder population according to the Bologna criteria represents only a 5 to 10% of patients in most assisted reproduction clinics, which creates logistic problems when performing a prospective study with sufficient power. Although the patients were not randomized, the two populations had similar baseline characteristics, which made it possible to compare IVF outcomes between the groups.

Obviously, the findings of this study should be confirmed by other groups in larger randomized trials, which could shed light on the specific aspect of the sensitizing effect of TT pre-treatment in COH of poor responder patients according to the Bologna criteria.

## CONCLUSIONS

Despite moderate evidence regarding the efficacy of TT as an adjuvant treatment in ovarian stimulation, this study suggests that the potential beneficial mechanism of TT in poor responder patients may be based on increasing the antral follicle sensitivity to the action of gonadotrophin treatment. FORT is an excellent tool to demonstrate this.
